# Characteristics of preoperative steroid profiles and glucose metabolism in patients with primary aldosteronism developing adrenal insufficiency after adrenalectomy

**DOI:** 10.1038/s41598-021-90901-4

**Published:** 2021-05-27

**Authors:** Xiao Wang, Daniel A. Heinrich, Sonja L. Kunz, Nina Heger, Lisa Sturm, Olaf Uhl, Felix Beuschlein, Martin Reincke, Martin Bidlingmaier

**Affiliations:** 1 Medizinische Klinik und Poliklinik IV, Klinikum der Universität München, Ziemssenstraße 1, 80336 Munich, Germany; 2 Division of Metabolic and Nutritional Medicine, Dr. von Hauner Children’s Hospital, Lindwurmstr. 4, 80337 Munich, Germany; 3 Klinik für Endokrinologie, Diabetologie und Klinische Ernährung, Rämistrasse 100, 8091 Zurich, Switzerland

**Keywords:** Endocrinology, Adrenal gland diseases, Scientific data

## Abstract

Treatment of choice in patients with unilateral aldosterone producing adenoma (APA) is adrenalectomy. Following surgery, most patients retain normal adrenal function, while some develop adrenal insufficiency (AI). To facilitate early detection and treatment of AI, we aimed to identify variables measured pre-operatively that are associated with post-operative AI. Variables obtained from 66 patients before and after surgery included anthropometrical data, clinical chemistry, endocrine work-up. LC–MS/MS steroid hormone profiles from tests before surgery (ACTH-stimulation, saline infusion, dexamethasone suppression) were obtained. Based on 78 variables, machine-learning methods were used in model fitting for classification and regression to predict ACTH-stimulated cortisol after surgery. Among the 78 variables, insulin concentration during pre-operative oral glucose tolerance test (OGTT) correlated positively, and dexamethasone suppressed glucocorticoids correlated negatively with ACTH-stimulated cortisol after surgery. Inclusion of LC–MS/MS measurements allowed construction of better models associated with the occurrence of AI in the training data, but did not allow reliable prediction in cross-validation. Our results suggest that glucocorticoid co-secretion (low insulin during pre-operative OGTT and insufficient suppression of glucocorticoids following dexamethasone) are correlated with the development of post-operative AI. Addition of steroid profiles improved the accuracy of prediction, but cross validation revealed lack of reliability in the prediction of AI.

## Introduction

Primary aldosteronism (PA) is an adrenal disease characterized by inappropriate autonomous production of aldosterone and consecutively suppressed renin concentrations^1–4^. The prevalence of PA ranges between 5 and 15% in the general hypertensive population and between 14 and 21% in resistant hypertension, respectively^5, 6^. Today, PA is considered the leading cause of secondary hypertension that can be cured by surgery^7^. Early diagnosis and treatment are important, since PA patients have a higher risk of cardiovascular events and target organ damage than normal hypertensives^8–10^. Aldosterone-producing adenoma (APA) and bilateral adrenal hyperplasia (BAH) are the two most common forms of PA^11^, accounting for 40% and 60% of the cases, respectively^12^. Therapeutic options differ depending on the subtype. Targeted medical treatment with mineralocorticoid receptor antagonists (MRA) is used in patients with BAH, while surgical removal of the aldosterone producing tumor by laparoscopic unilateral adrenalectomy is recommended in patients with unilateral disease^13, 14^. After surgery, most patients with APAs achieve complete or partial biochemical remission, and improvements in blood pressure control^15^. However, besides the intended reduction in mineralocorticoids, in some patients unilateral adrenalectomy results in impaired glucocorticoid secretion^16^.

We recently have reported prevalence and clinical characteristics of insufficient glucocorticoid production in a subgroup of APA patients following unilateral adrenalectomy, resulting in transient or even prolonged adrenal insufficiency (AI) and rarely to a life-threatening adrenal crisis. As reported recently, twenty-seven percent of the cohort developed postoperative AI, while those with severe AI (stimulated cortisol < 13.5 µg/dL) needed significantly longer hydrocortisone replacement therapy than those who were classified to have moderate AI (stimulated cortisol 13.5 to 17 µg/dL) (median in days (25th, 75th percentile): severe AI: 353 (294, 476) vs moderate AI: 74 (32, 293); p = 0.016)^17^. A likely reason for the occurrence lays in the fact that some APAs also co-secrete glucocorticoids, leading to minimal suppression of pituitary ACTH secretion and thereby, glucocorticoid production from the contralateral adrenal^18^. Consequently, after surgical removal of the APA, the suppressed contralateral adrenal would be unable to produce sufficient glucocorticoids until the recovery of the hypothalamic–pituitary–adrenal axis. Since late diagnosis or unawareness of AI would potentially endanger patients, early identification of those at risk to develop AI is desirable. However, in our recently published study we could not identify clinical or biochemical variables allowing to preoperatively predict the risk to develop AI after surgery^17^. Our hypothesis for the present study was that extending standard biochemical testing by dynamic endocrine tests combined with LC–MS/MS-based steroid profiling would allow us to better characterize the adrenal capacity of the patients, and thereby to predict the individual risk for development of AI following adrenalectomy. Indeed, LC–MS/MS based steroid profiling has recently been demonstrated to be useful in identification of subtypes of PA^19–22^. Our objectives were (a) to identify variables before surgery that correlate to cortisol production after surgery; (b) to determine the utility of LC–MS/MS steroid profiling before surgery to predict AI after surgery; and (c) to cross-validate the performance of a prediction model established based on our data.

## Results

### Single variable analysis

**Figure 1:**
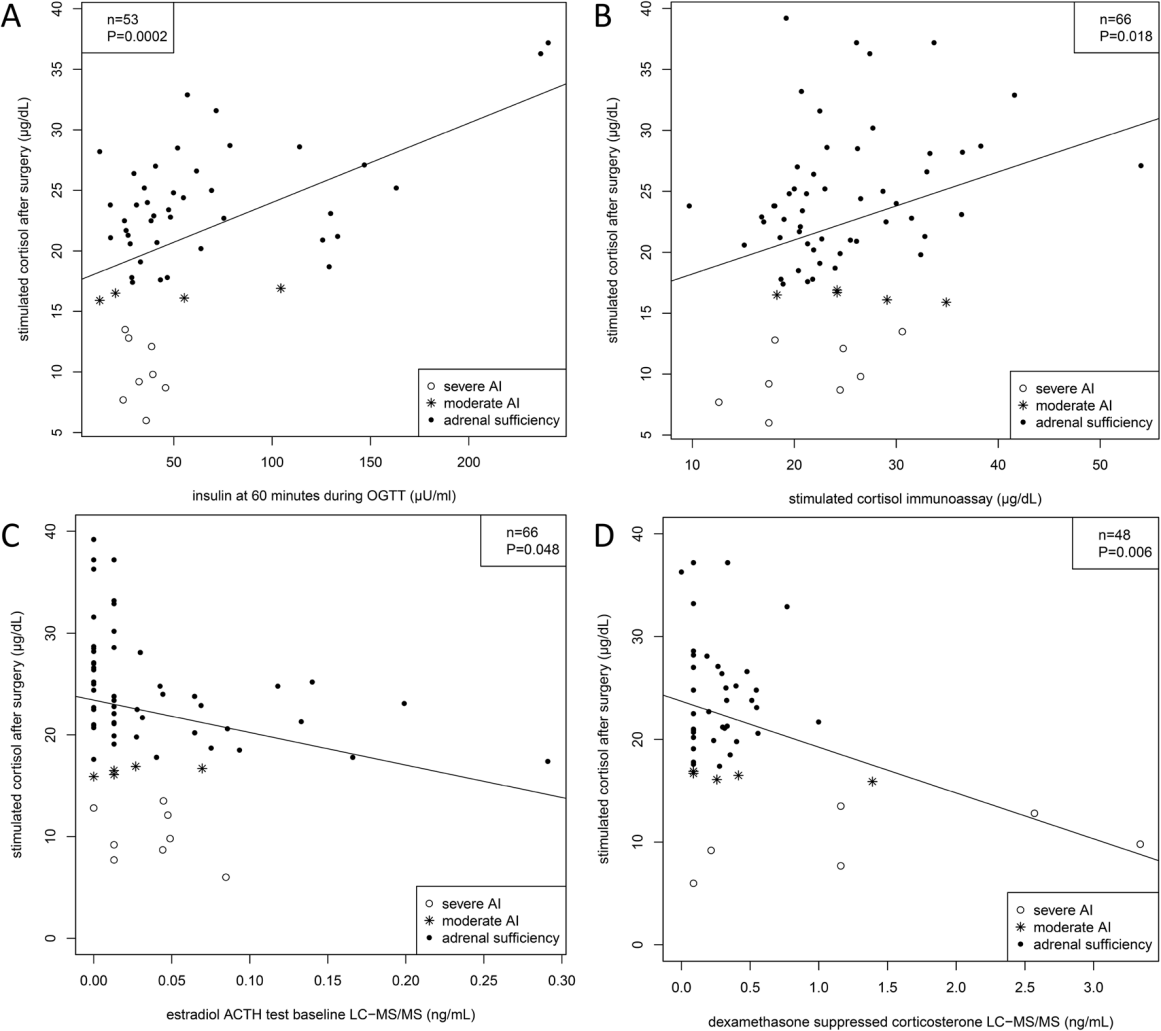
Single variables correlating with post-operative stimulated cortisol. Pre-operative insulin at 60 min during oral glucose tolerance test (OGTT) is positively correlated with post-operative stimulated cortisol (**A**). Pre-operative stimulated cortisol is positively correlated with post-operative stimulated cortisol (**B**). Pre-operative ACTH test baseline estradiol is negatively correlated with post-operative stimulated cortisol (**C**). Pre-operative dexamethasone suppressed corticosterone is negatively correlated with post-operative stimulated cortisol (**D**).

We identified 9 out of 78 variables collected before surgery which exhibited statistically significant (a = 0.05) correlation with stimulated cortisol after surgery. Two of the variables were cortisol measured by immunoassay at distinct time points (pre-operative ACTH stimulated cortisol (Fig. 1B) and salivary cortisol at 20:00). Three variables were steroids measured by LC–MS/MS (baseline estradiol (Fig. 1C), corticosterone (Fig. 1D) and 21-deoxycortisol following dexamethasone suppression). The other four variables were related to glucose metabolism: glucose and insulin at 60 min (Fig. 1A) as well as insulin at 120 min during OGTT and hemoglobin A1C (Hba1c). Notably, age was not correlated to stimulated cortisol after surgery, but higher estradiol values correlated with lower post-operative stimulated cortisol values as 61% of AI patients were females. In order to further confirm the significance of the correlations observed, we adjusted the significance level for the number of variables (a = 0.05/78 variables) to avoid accumulation of false positive rejections of the null hypothesis. After this correction, insulin at 60 min during OGTT still significantly correlated with ACTH stimulated cortisol after surgery (Fig. 1A).

**Figure 2:**
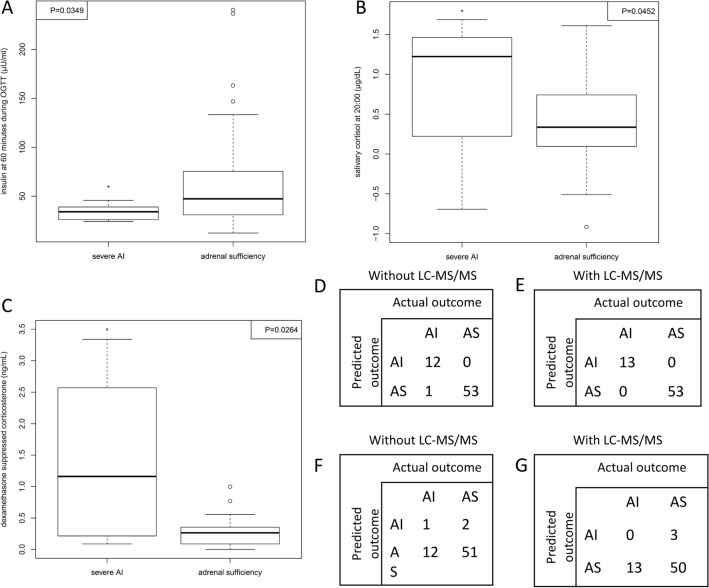
Boxplots of differences between severe AI and adrenal sufficiency groups in individual variable (**A**–**C**); the classification of gradient tree boosting model in training data (**D,E**) and cross validation (**F,G**). *Represent p value smaller than 0.05, namely significant difference. Boxplot of pre-operative insulin at 60 min during oral glucose tolerance test (OGTT) (**A**). Boxplot of salivary cortisol at 20:00 (**B**). Boxplot of dexamethasone suppressed corticosterone (**C**).

Comparing patients from the sAI and AS groups, a significant difference between the groups was observed for 4 variables: salivary cortisol at 20:00 (sAI (mean (SD): 2.988 (1.802) ng/mL vs. AS 1.7102 (0.977) ng/mL, p = 0.0452, Fig. 2B), dexamethasone suppressed corticosterone (sAI (mean (SD): 1.42 (0.28) ng/mL vs. AS 1.29 (0.22) ng/mL, p = 0.0264, Fig. 2C), insulin at 60 min during OGTT (sAI (mean (SD): 33.55 (7.72) μIU/mL vs. AS 67.65 (55.03) μIU/mL, p = 0.0349, Fig. 2A), and baseline HbA1c (sAI (mean (SD): 4.95 (0.41) % vs. AS 5.38 (0.56) %, p = 0.0212). ROC analysis showed area under the curve (AUC) values of 0.738 for insulin at 60 min and 0.709 for HbA1c, supporting their ability to distinguish between sAI and AS groups. HOMA-IR and HOMA-ß were calculated for the patient cohort. sAI patients showed a tendency towards lower HOMA-ß and higher HOMA-IR values in comparison to the AS group, although the difference did not reach statistical significance. Median and interquartile range of all variables in the three groups are provided in Supplement Table S2.

### Multiple-variable analyses

**Figure 3:**
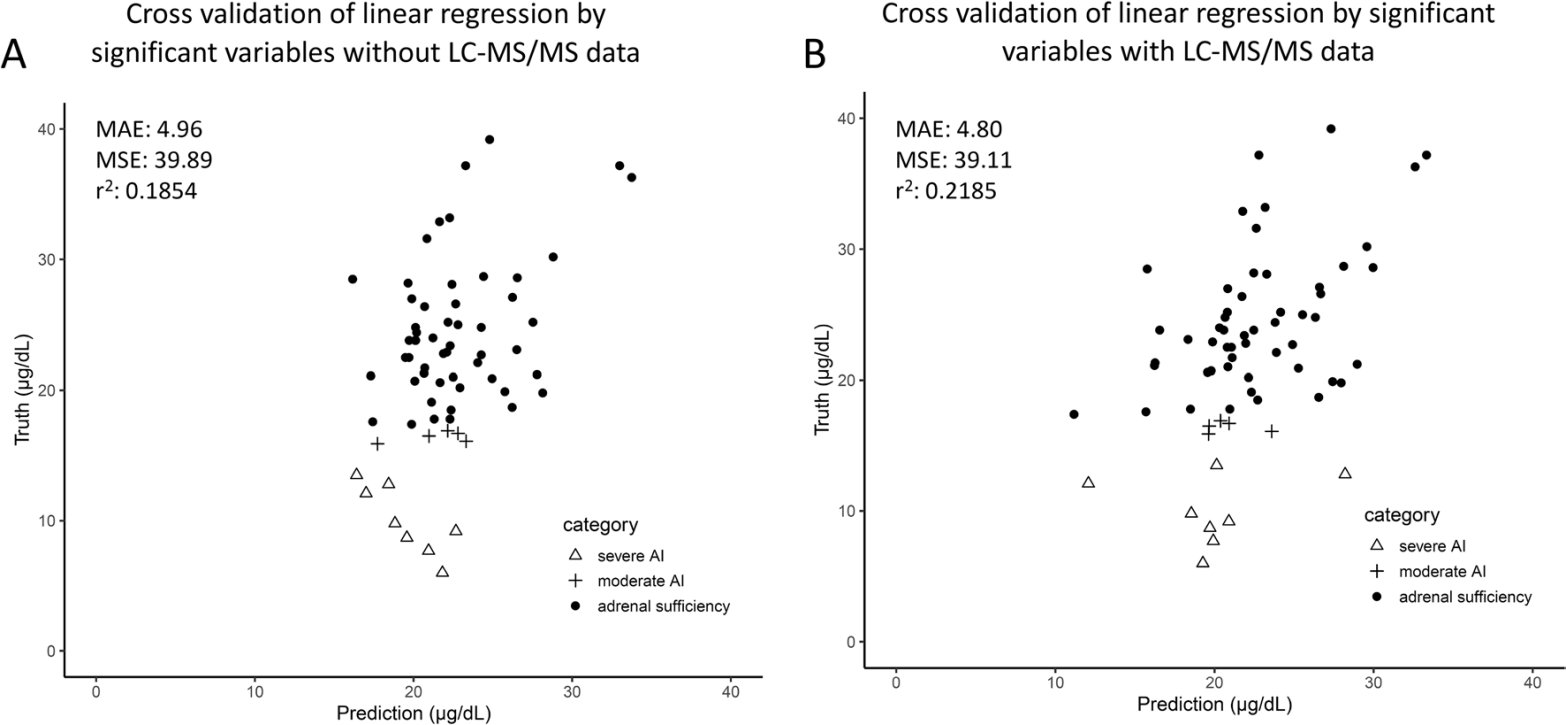
Plot of the true value and predicted value by linear regression model without (**A**) and with (**B**) LC–MS/MS in cross validation.

In order to investigate associations between preoperative variables and the occurrence of postoperative AI, we built linear regression models with the significant variables that we found in the single variable analysis. As mentioned above, nine variables fell into this category. Of those, two could not be used because more than 20% of the data were missing for steroid measurements by LC–MS/MS after dexamethasone suppression test (most were undetectably low). From the remaining seven variables, we excluded another three because they had no significant contribution to the model. The remaining four variables were insulin during OGTT at 60 min, salivary cortisol at 20:00, baseline cortisone and baseline estradiol during ACTH test. According to standard coefficients (Table 1), the strength of the effects was highest for insulin during OGTT at 60 min, followed by salivary cortisol at 20:00, baseline cortisone and baseline estradiol. The R squared between the true and the predicted value in LOOCV was 0.2185 (MAE 4.80, MSE 39.11, Fig. 3B). Leaving out data from LC–MS/MS measurements and using only insulin at 60 min during OGTT and salivary cortisol at 20:00 led to a further drop in R squared between the true and the predicted values in LOOCV to 0.1854 (MAE 4.96, MSE 39.89 (Fig. 3A)). The associations between the predicted values from the linear regression model and the true values can be seen in Fig. 3B.

**Table 1:** Coefficients, standard coefficients and p-values of coefficients of each variable in the linear regression model.

	Coefficients	Standard coefficients	p-value (coefficients)
(Intercept)	16.38388	6.709e−17	1.0000
Insulin at 60 min during OGTT	0.06450	4.444e−01	7.39e−05***
Salivary cortisol at 20:00	− 1.38434	− 2.339e−01	0.0282*
Cortisone ACTH test baseline	0.27062	2.332e−01	0.0291*
Estradiol ACTH test baseline	− 29.65508	− 2.266e−01	0.0322*

*p-value <0.05; ***p-value < 0.001.

As mentioned above, the other 69 variables recorded before surgery had no significant correlation to outcome as a single variable. We next explored if a combination of multiple variables could improve prediction and applied machine learning methods. Due to our limited sample size and the large amount of variables, we mainly used the methods with embedded feature selection, such as tree-based methods and regressions with regularization. 7 different statistical methods, namely lasso regression, ridge regression^23, 24^, random forest^25^, decision tree^26^, model-based boosting^27^, gradient tree boosting^28^ and xgboost^29^, were performed to identify the best model. We integrated cross validation (LOOCV) to evaluate the predictive capacity of each model established by the training data on a new observation. In the following, we only report analyses using xgboost and model-based boosting since these methods outperformed the others by far.

#### Classification of patients regarding adrenal insufficiency and sufficiency

For classification analysis, we combined mAI and sAI patients into one “AI group” (n = 13, eight females and five males) and compared this group to the AS group (n = 53, 28 females, 25 males). Without the 15 steroid hormones measured by LC–MS/MS on two occasions included in the variables, xgboost correctly classified 100% of AS patients and 92.3% (12 out of 13) of AI patients by 33 variables (e.g. age, insulin at 60 min during OGTT and salivary cortisol at 20:00) in training data (Fig. 2D). Adding the steroid profiles and using all 63 variables, 100% patients were correctly classified into both groups in the training data (Fig. 2E). However, the majority of AI patients were misclassified in cross validation (Fig. 2F,G). Therefore, the xgboost models seem to exhibit over-fitting to the training data.

#### Prediction of ACTH stimulated cortisol after surgery

**Figure 4:**
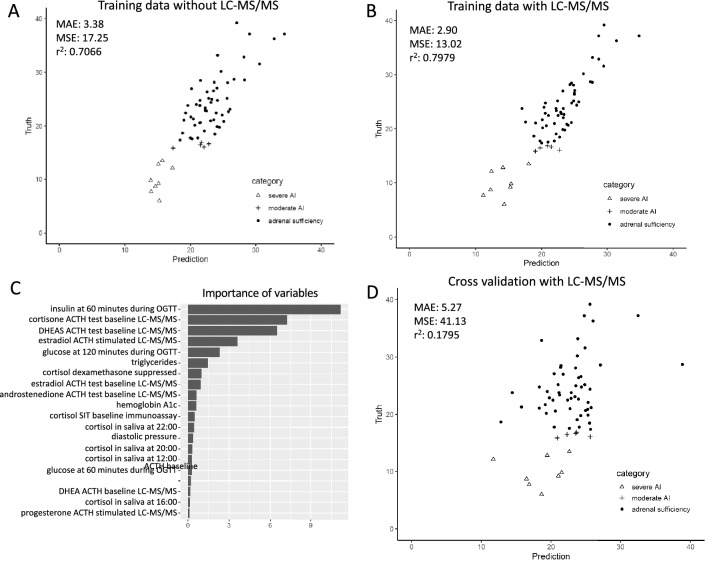
Plot of the true value and predicted value by model-based-boosting model without (**A**) and with (**B**) LC–MS/MS in training data. Importance of variables in model-based-boosting model (**C**). Plot of the true value and predicted value by model-based-boosting model with LC–MS/MS in cross validation (**D**).

To further evaluate if the combination of variables obtained before surgery can be used to reliably predict adrenal status after surgery, we applied the 7 statistical methods that were mentioned above. Regression model-based boosting achieved the best results in the training data. Without the 30 LC–MS/MS based variables, the adjusted R-squared between the true and the predicted values in training data was 0.7066 (mean absolute error (MAE) 3.38; mean squared error (MSE) 17.25 (Fig. 4A)). When the 30 variables from LC–MS/MS based steroid measurements were included, the adjusted R-squared increased to 0.7979 (MAE 2.90; MSE 13.02 (Fig. 4B)). Analysis of the importance of the individual variables for the model by permutation (Fig. 4C) revealed that insulin at 60 min during OGTT was the most important feature in the model, followed by baseline cortisone and baseline DHEAS during ACTH test.

In contrast to the performance of the models in the training data, in the cross-validation R-squared dropped to 0.1795 (MAE 5.27, MSE 41.13; Fig. 4D). Accordingly, the model including the variables was not better than a model using a constant number (MAE 5.35, MSE 50.27). This indicates that even with inclusion of the LC–MS/MS based steroid profiles, the model-based boosting models generated from the training data cannot be extrapolated to other data.

## Discussion

Our study identified several single features before surgery that significantly correlated with adrenal cortisol production after surgery, and also correlated with ACTH stimulated cortisol after surgery in linear regression models. In particular, peak insulin secretion during OGTT before surgery correlated positively with ACTH stimulated cortisol after surgery. In addition, a greater suppression of glucocorticoids by dexamethasone before surgery also was associated with higher stimulated cortisol after surgery. Although age and sex affect circulating concentrations of some steroids, they had no impact in any correlation analysis. We also demonstrated that adding information from steroid profiles determined by LC–MS/MS to conventional laboratory data could improve both, regression with ACTH stimulated cortisol after surgery and accuracy of classification of patients in the AI and AS groups in our data. However, when we used LOOCV to test the predictive capacity of various models established by our data, poor sensitivity was revealed. This indicates that—although additional variables from the LC–MS/MS profiles significantly improved the models in the training data—it is not possible to reliably predict adrenal function post-surgery based on the preoperative variables available in our study.

The positive association of insulin secretion during OGTT before surgery to ACTH stimulated cortisol post-surgery was a consistent observation in our analyses. Insulin was also the most important variable in the regression model-based boosting analysis to predict adrenal status post-surgery in our training data (Fig. 4B) and had the largest influence in the linear regression model with the best cross validation (Table 1). ROC analysis showed that the AUC for insulin at 60 min and HbA1c were best to distinguish between sAI and AS groups (Supplemental Table S2). Co-secretion of glucocorticoids from aldosterone producing adenomas has been demonstrated^18^. It is well-known that long-term exposure to excess glucocorticoids lead to the development of higher hepatic glucose production and decreased insulin-dependent glucose uptake into peripheral tissues, such as muscles—causing insulin resistance in a reactive hyperinsulinemic state^30, 31^. However, in addition to their effect on glucose sensitivity, glucocorticoids are also known to acutely inhibit insulin secretion from pancreatic ß-cells^30, 32^. Furthermore, glucocorticoids decrease biosynthesis of insulin by reducing ATP/ADP ratio, while inducing ß-cells apoptosis directly and indirectly. Even in in vivo experiments with transgenic mice overexpressing the glucocorticoid receptor in ß-cells it was shown that initial insulin secretion decreased during a glucose load^33, 34^. In humans, a large study from Japan showed an association between higher serum cortisol levels and decreased insulin secretion (lower HOMA-ß values) in a general population^35^. These findings support that ß-cell function and insulin secretion are reduced by elevated levels of glucocorticoids. Kamba et al. argue that—although high serum glucocorticoid concentrations are positively correlated with insulin resistance and with a compensatory increase of insulin secretion long term—acute suppression of ß-cell function may constitute for another aspect of glucocorticoid effects on glucose metabolism. In this setting, glucocorticoids cause suppression of insulin secretion. When glucocorticoid concentrations further increase over time to consistently elevated levels, insulin resistance develops. Therefore, insulin resistance as well as ß-cell dysfunction lead to impaired glucose metabolism and the development of diabetes^35^. Our observation of lower peak insulin values during OGTT might reflect this aspect of the interplay between glucocorticoids and insulin secretion. In accordance, sAI patients tended to have lower HOMA-ß values, indicating suppressed ß-cell function. Thus, the lower 60 min. insulin concentrations during OGTT in the patients with higher glucocorticoids (which are at risk to develop AI post-surgery) do not contradict the general understanding on glucocorticoid effects on glucose/insulin homeostasis but might just reflect an early effect of glucocorticoids on the insulin secretion in our patient subgroup. This would explain why patients with lower peak insulin values could have experienced greater glucocorticoid exposure. This would be in line with our observation that dexamethasone suppression of glucocorticoids was less efficient, and that salivary cortisol at night was higher in the AI group. One could speculate about a mechanism where greater glucocorticoid exposure before surgery leads to a stronger suppression of glucocorticoid secretion from the contralateral adrenal, increasing the risk to develop transient adrenal insufficiency following the removal of the APA. Postoperative histopathological tumor sizes were also compared among the groups. PA patients with sAI and mAI tended to have slightly larger tumors, however not reaching statistical significance (median in mm: sAI: 17 (12.5; 20); mAI 15 (15; 25); AS 11 (8; 15), p = 0.096).

The use of LC–MS/MS based steroid profiles has contributed significantly to our understanding of adrenal disease^18, 36–40^. Two recent studies^20, 41^ have demonstrated that—by using slightly different LC–MS/MS based profiles of 15 steroids—it was possible to correctly classify the subtype of PA. Apart from allowing distinguishing between BAH and APA, there were also associations of the steroid profiles obtained by LC–MS/MS with adenoma genotype^21^. 18-hydroxycortisol and 18-oxocortisol have been shown to be helpful to distinguish BAH and APA^41^. Unfortunately, our steroid profiles did not include these two steroids and limitations from sample volume did not allow a separate analysis.

Until today, no data have been published on the potential value of pre-surgical LC–MS/MS based steroid profiles to predict the occurrence of AI after surgery. In our study, the steroid profiles obtained at any of the three different time points before surgery alone were not sufficient to establish associations with AI following surgery. We therefore combined the three steroid profiles together with 33 conventional parameters for feature selection, and subsequently compared models for correlation, classification and prediction without and with inclusion of the steroid profiles. We found that LC–MS/MS based steroid profiles can significantly improve the correlation models for ACTH stimulated cortisol post-surgery, and also help to correctly classify the AI and AS groups. Furthermore, inclusion of LC–MS/MS based steroid profiles reduced the error between predicted and true values in cross validation of the linear regression models.

However, although the model with LC–MS/MS data in classification achieved 100% accuracy in our training data, the majority of cases in the AI group was misclassified as AS in the cross validation. A discrepancy between the performance of models in the training data and the prediction in cross validation is frequently observed. A likely explanation is that such models tend to be over-fitted to the training data, and do not correctly embody the characters of the data^42^. Consequently, predictive potency in the training data can be optimistically biased. Imbalance in the size of the groups can also dramatically decrease the power of statistical methods^43^. In our case, the bigger group with 53 AS patients tends to dominate the classification models, and characters of the smaller group with 13 AI patients might not be taken up by the model.

Unfortunately, since the extended set of dynamic tests performed in our cohort is not part of clinical routine elsewhere, and patients undergoing adrenalectomy still are rare, it was impossible to get an independent new dataset for testing the models. However, we followed a standard approach to evaluate the performance of models by statistical techniques established for cross validation in the absence of independent data sets. The extent of the prediction in cross validation matching with the true values is the key criterion. Only when the prediction in cross validation is better than a featureless model, a model can be assumed to appropriately reflect existing relationships between the predictors and the target in the dataset. In addition to using all features, we also used two to seven of the top features only to build xgboost models to avoid overfitting. Indeed, using only the top four features in the xgboost, allowed correct classification of two more AI patients. However though slightly better, this is still not good enough to predict AI in a clinical setting. This finding supports our hypothesis that adding more variables can cause problems with overfitting. The fact that in our analysis a simplified linear model with the four significant variables was superior to a sophisticated model-based-boosting model with all the variables implies that including more variables must not necessarily lead to improved prediction but might just increase the noise in the data. Instead of just adding more variables, a guided selection of variables based on background knowledge about physiological relationships might be more appropriate to improve the models.

In summary our findings support the notion that glucocorticoid co-secretion from aldosterone-producing adenomas might be a risk factor for the development of adrenal insufficiency after adrenalectomy. Based on our findings, a prediction of the risk of AI during the pre-operative period still is not possible with enough reliability. Because of the elevated risk to develop transient adrenal insufficiency after unilateral adrenalectomy, we already do in our institution, and would generally recommend that clinicians should perform ACTH stimulation tests in all subjects with APAs after surgery, as AI is a potentially life-threatening condition. Better characterization of glucose metabolism before surgery could be important to understand the association. Furthermore, our study demonstrates that correlation- and classification models were improved by inclusion of data from LC–MS/MS based steroid profiles. In the training data, it was also possible to develop algorithms for accurate prediction of the adrenal function after surgery based on steroid profiles obtained before surgery. However, we also realized that our prediction models from the training data failed correct classification in cross-validation studies. This exemplifies that—while new technologies including the measurement of steroid profiles by LC–MS/MS allow increasing the number of variables—confirmation of the predictive value of adding more variables through cross-validation is crucial.

## Materials and methods

### Patients

In this study, we retrospectively analyzed blood samples obtained from 66 patients (30 males/36 females, age range 24–73 years) who consented to be included in the prospective cohort of the German Conn’s registry and had a confirmed diagnosis of APA based on aldosterone-to-renin ratio (ARR), saline infusion testing (SIT) and underwent non-ACTH stimulated adrenal vein sampling (AVS) for subtyping. Clinical characteristics of the patients’ cohort as well as details of the screening and confirmatory tests have recently been described^17^. From the original cohort of 100 patients, 66 underwent pre- and postoperative operative ACTH stimulation testing and were therefore included in the current analysis. Patients were classified as lateralized according to standard imaging and biochemical criteria (AVS: selectivity index > 2, lateralization index > 4) and underwent unilateral adrenalectomy at our institution from August 2014 to December 2018. All patients also received a preoperative 1 mg overnight dexamethasone suppression test (cut-off serum cortisol: > 1.8 µg/dL), measurement of late-night salivary cortisol (cut-off < 1.5 ng/mL) and 24-h collection of urinary free cortisol (cut-off < 83 µg/L). An oral glucose tolerance test (OGTT, 75 g) with insulin and glucose measurements at baseline and after 60 and 120 min was performed in patients who did not have a diagnosis of diabetes mellitus type 1 or 2. In total, three patients had a history of diabetes mellitus type 2. All blood, urine and saliva samplings and dynamic tests were performed according to the standards of the German Conn registry^44^. Informed consent was obtained from all study participants. The study was designed in agreement with the Declaration of Helsinki and approved by the Ethics Committee of the Medical Faculty of the Ludwig-Maximilians-University, Munich.

### Definition of AI

Preoperative ACTH stimulation tests were performed one before adrenalectomy. Patients underwent postoperative testing on the day of discharge from the hospital (usually on the 4th or 5th day after surgery). Tests started at 8 AM after resting in a calm environment for at least 30 min. Serum cortisol levels were measured before and 30 min after intravenous application of 0.25 mg of 1‒24 ACTH (Synacthen). Pre- and postoperatively, adrenal function was classified according to the outcome of the ACTH stimulation test. Since the patients’ treatment was based on our routine cortisol assay, we also used this assay for patient classification. Adrenal sufficiency (AS) was defined by ACTH stimulated serum cortisol ≥ 17 µg/dL, moderate adrenal insufficiency (mAI) between 13.5 and 17 µg/dL, and severe adrenal insufficiency (sAI) by stimulated serum cortisol ≤ 13.5 µg/dL. Postoperatively, 8 of the 66 patients were classified as sAI; 5 patients as mAI and 53 patients as AS. Since the group size was highly unbalanced, for all multiple-variable analyses we combined both, sAI and mAI, as the “AI group” (n = 13).

### Variables

Overall, we included 78 patient variables obtained before surgery in our analysis (see Supplemental Table S1). The variables included anthropometric data (e.g. sex and age), parameters routinely obtained during physical examination (e.g. body mass index (BMI) and blood pressure (RR)), but also clinical chemistry and hormone assessments from the routine laboratory at baseline (e.g. cortisol and ACTH by immunoassay, potassium) and following dynamic tests (e.g. ACTH stimulated cortisol). In addition, we measured 15 adrenal steroids by LC–MS/MS from samples taken before surgery at baseline and after ACTH stimulation and after dexamethasone suppression. The 15 steroids were aldosterone, cortisol, cortisone, corticosterone, 11-deoxycortisol, 21-deoxycortisol, dehydroepiandrosterone (DHEA), DHEA-sulfate (DHEAS), estradiol, testosterone, androstenedione, 11-deoxycorticosterone, dihydrotestosterone, 17-hydroxyprogesterone and progesterone. HOMA-IR (homeostatic model assessment–insulin resistance) was calculated using this equation: HOMA IR = fasting insulin (µU/mL) × fasting glucose (mg/dL)/405. HOMA-ß (homeostatic model assessment − ß-cell function) was calculated using this equation: HOMA-ß = 360 × fasting insulin (μU/mL)/(fasting glucose (mg/dL) − 63). Insulinogenic Index was calculated using this equation: IGI = Insulin at 60 min (μU/mL) – fasting insulin (μU/mL)/(glucose at 60 min (mg/dL) – fasting glucose (mg/dL)).

### Assay methods

For clinical workup, hormone concentrations were measured by routine immunoassay methods (active renin concentration, aldosterone, ACTH, cortisol all by Liaison, Diasorin, Saluggia, Italy). Steroid measurements by LC–MS/MS were performed using a commercially available kit (Chromsystems, Darmstadt, Germany) according to the manufacturer’s instructions. In brief, solid phase extraction (SPE) with 500 µL of samples, calibrator solutions and quality control samples was performed. An internal standard mix (72,044, Chromsystems), containing deuterated analogs of all measured steroids, was added to the samples and each of the calibrator solutions and serum controls. The SPE procedure included two washing steps and an elution step with 500 µL elution buffer (72,033, Chromsystems). After SPE, the extract was evaporated to dryness and dissolved in reconstitution buffer (72,006, Chromsystems). The kit included serum calibrator set (72,038–72,039, Chromsystems) provided seven different concentration levels for the calibration and three different concentration levels of serum controls (0341–0347, Chromsystems). Additionally, three quality controls from an external supplier (Liquicheck, BioRad) were measured. The steroids were divided in two panels that were measured separately with an injection volume of 20 µL by gradient elution with mobile phase A (72,011, Chromsystems) and B (72,002, Chromsystems). Chromatographic separation and detection were performed with 1290 Infinity II HPLC System (Agilent, Waldbronn, Germany) coupled to a QTrap 6500 + tandem mass spectrometer (Sciex, Darmstadt, Germany). Electrospray ionization in positive and negative mode was used for ionization and measurement mode was multi reaction monitoring. Data analysis was performed using Analyst 1.6.3 Software (Sciex, Darmstadt, Germany).

### Statistical methods

Statistical analyses were conducted using R (version 3.6.1). R packages “MASS”, “tidyverse”, “glmnet”, “mice”, “DMwR”, “caret”, “randomForest”, “mlr”, “DataExplorer” and “corrplot” were required for analysis. Correlation coefficient was used to assess the correlation between variables obtained before surgery and ACTH stimulated cortisol after surgery. Analysis of variance or Kruskal–Wallis-Test was used to assess the difference between two groups. The equality of variance was tested by Levene's test and Shapiro–Wilk test was used in test of normality. When required, data were logarithmically transformed. Model-based boosting was used in model fitting for regression. Gradient Tree boosting was used for classification. Leave-one-out cross validation (LOOCV) was integrated for the estimation of performance of models. Due to the small sample size we chose to use LOOCV instead of the commonly used k-fold cross validation methods. Permutation importance was used to calculate the importance of the variables^45^. Missing data were imputed by K-nearest neighbor’s algorithm for multiple-variable analyses. Variables containing more than 20% missing values were excluded. This applied only to 15 steroid concentrations after dexamethasone suppression test measured by LC–MS/MS (details see Supplemental Table S1). Therefore, from the 78 variables, only 63 were available for multiple-variable analyses. Steroid hormone concentrations below the limit of quantification (LoQ) of the respective assay methods were replaced by 50% of the corresponding LoQ.

## Supplementary Information

Supplementary Table S1.

Supplementary Table S2.

## Data Availability

The datasets generated during and/or analyzed during the current study are available from the corresponding author on reasonable request.

## Abbreviations

ACTH: Adrenocorticotropic hormone
AI: Adrenal insufficiency
APAs: Aldosterone-producing adenomas
ARR: Aldosterone-to-renin ratio
AS: Adrenal sufficiency
AVS: Adrenal vein sampling
BAH: Bilateral adrenal hyperplasia
DHEA: Dehydroepinandrosterone
Hba1c: Hemoglobin A1C
HOMA-IR/HOMA-ß: Homeostatic model assessment–insulin resistance/ß
LC–MS/MS: Liquid chromatography with tandem mass spectrometry
LOOCV: Leave-one-out cross validation
LoQs: Limits of quantifications
MAE: Mean absolute error
MSE: Mean squared error
OGTT: Oral glucose tolerance test
PA: Primary aldosteronism
SIT: Saline infusion test
SPE: Solid phase extraction
